# Deletion of *pic* results in decreased virulence for a clinical isolate of *Shigella flexneri* 2a from China

**DOI:** 10.1186/1471-2180-13-31

**Published:** 2013-02-08

**Authors:** Junqi Zhang, Lisheng Qian, Yang Wu, Xia Cai, Xueping Li, Xunjia Cheng, Di Qu

**Affiliations:** 1 Department of Medical Microbiology and Parasitology, School of Basic Medical Sciences, Fudan University, No. 138 Yixueyuan Road, Shanghai, 200032, China; 2 Key Laboratory of Medical Molecular Virology of Ministries of Education and Health, Institute of Medical Microbiology and Institutes of Biomedical Sciences, School of Basic Medical Sciences, Fudan University, No. 138 Yixueyuan Road, Shanghai, 200032, China

**Keywords:** *Shigella flexneri*, Multiplex PCR, Clinical isolates, *Pic* gene, HeLa cell gentamicin protection assay, Mouse sereny tests

## Abstract

**Background:**

*Shigella* is a major pathogen responsible for bacillary dysentery, a severe form of shigellosis. Severity of the disease depends on the virulence of the infecting strain. *Shigella* pathogenicity is a multi-gene phenomenon, involving the participation of genes on an unstable large virulence plasmid and chromosomal pathogenicity islands.

**Results:**

A multiplex PCR (mPCR) assay was developed to detect *S. flexneri* 2a from rural regions of Zhengding (Hebei Province, China). We isolated and tested 86 strains using our mPCR assay, which targeted the *ipaH*, *ial* and *set1B* genes. A clinical strain of *S. flexneri* 2a 51 (SF51) containing *ipaH* and *ial*, but lacking *set1B* was found. The virulence of this strain was found to be markedly decreased. Further testing showed that the SF51 strain lacked *pic*. To investigate the role of *pic* in *S. flexneri* 2a infections, a *pic* knockout mutant (SF301*-*∆ *pic*) and two complementation strains, SF301*-*∆ *pic*/pPic and SF51/pPic, were created. Differences in virulence for SF51, SF301*-*∆ *pic*, SF301*-*∆ *pic*/pPic, SF51/pPic and *S. flexneri* 2a 301 (SF301) were compared. Compared with SF301, both SF51 and SF301*-*∆ *pic* exhibited lower levels of Hela cell invasion and resulted in reduced keratoconjunctivitis, with low levels of tissue damage seen in murine eye sections*.* The virulence of SF301*-*∆ *pic* and SF51 was partially recovered *in vitro* and *in vivo* through the addition of a complementary *pic* gene.

**Conclusions:**

The *pic* gene appears to be involved in an increase in pathogenicity of *S. flexneri* 2a. This gene assists with bacterial invasion into host cells and alters inflammatory reactions.

## Background

Bacteria of the genus *Shigella* are fastidious Gram-negative organisms that cause an estimated 164.7 million cases of shigellosis annually worldwide, and are responsible for 1.1 million deaths [1]. Shigellosis is an acute intestinal infectious disease. Its symptoms range from mild watery diarrhea to a life-threatening dysenteric syndrome with blood, mucus and pus in stools [2-4]. The severity of the disease depends on the virulence of the infecting strain. Therefore, clinical diagnosis tests for Shigellosis should not only focus on the determination of the strain’s biochemical and serological types, but also on the determination of the strain’s virulence. Based on biotyping, the *Shigella* genus contains four species with 48 serotypes (including subgroups). In China, *Shigella flexneri* 2a (*S. flexneri* 2a) is the predominant subgroup [2].

To simultaneously, effectively, and rapidly detect the pathogen and determine its virulence, three chromosome- and plasmid-encoded virulence genes (*ipaH*, *ial*, and *set1B*) [3,5-7] were chosen to assist in the development of a multiplex PCR (mPCR) assay. *ipaH* is present on both the chromosome and on the large *Shigella* virulence plasmid. Therefore, *ipaH* is considered a stable PCR target for pathogen identification [8-11]. The *ial* gene is located in the cell-entry region of the large virulence plasmid that encodes an important part of the molecular machinery required for bacterial invasion and intracellular survival [4,12-14]. This region is bracketed by insertion-like (IS) elements IS100 and IS600, with a high tendency for automatic deletion [4,13,15,16]. Detection based on *ial* provides some information pertaining to bacterial virulence but can easily generate false negative results [4,17]. The *set1B* gene is located on pathogenicity island 1 (PAI-1) of the chromosome and encodes *Shigella* enterotoxin 1 subunit B. Enterotoxin 1 causes the watery phase of diarrhea in Shigellosis [6,18,19]. Studies have shown that *set1B* is present exclusively in *S. flexneri* 2a [6,18,19]. An mPCR system should be able to determine, in a single reaction, whether the genes related to pathogenesis of a particular *Shigella* strain are encoded on the chromosome or the plasmid, and also to determine the serotype of a particular strain [4,5].

The *S. flexneri* 2a *pic* gene, which is located at an unstable chromosomal site of *S. flexneri* 2a PAI-1, is spontaneously deleted at a low frequency [20]. Previous studies have shown that the *pic* and *set1B* loci are overlapping genes encoded on opposite strands, and *set1B* is within *pic*[21]. The Pic protein is a 116 kDa auto-transporter protein, secreted by the serine protease auto-transporter from members of the *Enterobacteriaceae* family [21,22]. To date, *pic* has only been found in enteroaggregative *Escherichia coli* (EAEC), uropathogenic *E. coli* (UPEC) and *S. flexneri* 2a. Pic has been shown to exhibit hemagglutination and mucinolytic activities *in vitro*[21-24]. However, it has also been shown that Pic is unable to elicit a cytotoxic effect in the HT29-C1 and HEp-2 epithelial cell lines [24,25].

The major aims of our study were to detect and determine the strain of the *Shigella* pathogen and determine its virulence. We also investigated whether attenuation of SF51 virulence correlated to the loss of *pic*, by constructing a *pic*-deleted mutant and two complementation strains.

## Methods

### Ethics

All procedures performed on mice were conducted according to national (Regulations for the Administration of Affairs Concerning Experimental Animals, China) and international guidelines (NIH Guide for the Care and Use of Laboratory Animals) and were approved by the Institutional Animal Care and Use Committee (IACUC) of Shanghai Medical College, Fudan University (IACUC Animal Project Number 20090601-QU).

### Bacterial strains, plasmids, media and growth conditions

Clinical isolates (*n* = 86) of *S. flexneri* were isolated from an epidemic site in Zhengding (Hebei Province, China). Serotyping of the strains was carried out by the Bacteriological Unit at Huashan Hospital (Shanghai, China). The *S. flexneri* 2a 301 (SF301; GenBank Accession No. AE005674) strain was provided by Dr. Jianguo Xu (Chinese Center for Disease Control and Prevention, Beijing, China). SF301 was isolated in 1984 from the Changping District of Beijing. The affected subject exhibited a severe acute clinical manifestation of Shigellosis. The complete genome of SF301 was sequenced and has since been used as a reference strain for *S. flexneri* 2a in China. *E. coli* ATCC 25922 was provided by Dr. Bijie Hu from Zhongshan Hospital (Shanghai, China). *E. coli* SM10 λpir and plasmid pSB890 were provided by Dr. Daoguo Zhou from Purdue University (West Lafayette, IN, USA). The pSC plasmid was modified from pREP4 (Qiagen, Hilden, Germany), which contains a p15A origin of replication and a kanamycin resistance gene. *E. coli* DH5α was purchased from Invitrogen (Carlsbad, CA, USA). *S. flexneri* and *E. coli* were grown at 37°C in Luria–Bertani (LB) medium (Oxoid, Wesel, Germany). All bacterial strains were grown on Salmonella–Shigella (SS) agar (Oxoid) before being transferred to an LB agar plate. Strains were then incubated overnight at 37°C, then stored at −20°C in LB broth containing 15% glycerol.

### Screening of clinical specimens by mPCR

The *ipaH*, *ial*, and *set1B* genes were detected by mPCR with primers designed according to the sequences of these genes in SF301 (Table 1) [3,5,7]. Clinical *S. flexneri* isolates (*n* = 86) were tested using mPCR. The mPCR mixture (20 μL) consisted of 1.8× PCR buffer (3 mM MgCl_2_, 130 μM dNTP; Invitrogen), 0.5 μM *ial* primer, 0.3 μM *ipaH* primer, 0.3 μM *set1B* primer, 1 U of Taq DNA polymerase (Invitrogen), and 10 μL of bacterial lysate. Thermal cycling conditions involved an initial denaturation step at 95°C for 5 min, followed by 30 cycles of 94°C for 1 min, 56°C for 1 min, and 72°C for 2 min, and a final extension step at 72°C for 7 min after the 30^th^ cycle.

**Table 1:** Sequences of oligonucleotide primers used in this study

**Target gene**	**Gene position on SF301 genome or virulent plasmid pCP301**	Primer*	**Primer sequence (5**^**′**^**→3**^**′**^**)**	**Length (bp)**
**Primers for detection of virulence-associated genes of *S. flexneri *by mPCR**				
*ipaH*	1422225–1422835 **	ipaH-F	CCTTGACCGCCTTTCCGATA	611
		ipaH-R	CAGCCACCCTCTGAGAGTACT	
*ial*	133550–133869***	ial-F	CTGGATGGTATGGTGAGG	320
		ial-R	CCAGGCCAACAATTATTTCC	
*set1B*	3069523–3069669**	set1B-F	GTGAACCTGCTGCCGATATC	147
		set1B-R	ATTTGTGGATAAAAATGACG	
**Primers for amplifying *int*, *orf30*, *sigA *and *pic *on PAI-1 of *S. flexneri *2a**				
*int*	3052736–3053998**	int-F	ATGGCACTGACTGACGCAAA	400
		int-R	TGCCGATAAAGGGGAAAACG	
*orf30*	3096187–3097975**	orf30-F	CTTATCACTGAGCGTCTGGT	1,102
		orf30-R	GTGAAATTCCTGCCTCAATA	
*sigA*	3060437–3064294**	sigA-F	AGTCATATTACAGGTGGATTAG	1,866
		sigA-R	TATACTCAGGGTTGCGTTTT	
*pic*	3067737–3070949**	pic-F	AGAACATATACCGGAAATTC	1,219
		pic-R	ACCCTGACGGTGAATAAACT	
**Primers for homologous recombination to construct *pic *knockout strain**				
upstream of *pic*	3067236–3067745**	up*pic*-F-NotI	AAGCGGCCGCCATAGCAGACTGGCCGGTCAACC	520
		up*pic*-R-XbaI	CCTCTAGAATGTTCTGATGTGGGGGTAAAGGGC	
downstream of *pic*	3071850–3072358 **	down*pic*-F-XbaI	CCTCTAGAATTCACTATGGATTCTCCATGAT	517
		down*pic*-R-BamHI	AAGGATCCCGTCGTCCGTCTGGCACC	
upstreamof *pic*	3066436–3072733**	Upup*pic*-F	GCTGAACTGC TGGAGCCGCT	1176
downstream of *pic*		Downdown *Pic*-R	CAGCGGCGAAATACTGTACC	
*pic* coding frame work	3067737–3070949**	*pic*-pSC-F-PfMlI	AAACCATCGAATGGATGCAGGACGATTTCGATGCCCCCGTAGAC	3,213
		*pic*-pSC-R-AclI	TTTAACGTTTCAGAACATATACCGGAAATTCGCGTT	

*F, forward primer; R, reverse primer.

**SF301 GenBank Accession No. AE005674.

***SF301 large virulent plasmid pCP301 GenBank Accession No. AF386526.

Underlined sequences represent restriction endonuclease sites.

### Plaque formation tests on HeLa cells

Twelve strains containing *ipaH*, *ial* and *set1B* were further tested to determine their virulence in HeLa cells (ATCC CCL-2) using a plaque formation test [26]. HeLa cells were grown in 24-well tissue culture plates until they formed semi-confluent monolayers. The culture medium used was RPMI1640 supplemented with 10% fetal calf serum (FCS), and 1% penicillin-streptomycin; and cultures were incubated at 37°C/5% CO_2_. Cells were washed three times with phosphate-buffered saline (PBS), and bacteria added to the semi-confluent HeLa cultures at a multiplicity of infection (MOI) of 100. After incubating at 37°C for 90 min, growth medium containing 5% (w/v) agar and 20 μg/mL gentamicin was poured into the 24-well plates, then incubated at 37°C/5% CO_2_ for 72 h. HeLa cells were inoculated with SF301 as a positive control, and with *E. coli* ATCC 25922 as a negative control.

### Sequence and analysis of virulence genes on PAI-1 of SF51

SF51 genomic DNA was extracted using a QIAamp DNA Mini Kit (Qiagen). PCR primers for amplification of *pic*, *sigA*, *int* and *orf30* from PAI-1 of the SF51 clinical isolate were designed according to the SF301 sequence. Amplicons were cloned into a pCR-XL-TOPO vector using a TOPO® XL PCR Cloning Kit (Invitrogen), and the inserts were sequenced by Sangon Biotech (Shanghai, China) Co. Ltd, then identified using the standard nucleotide basic local alignment search tool (BLASTn; NCBI).

### Construction of SF301-∆ *pic*

The upstream and downstream portions of *pic* were amplified by PCR. Primers up*pic*-F-NotI and up*pic*-R-XbaI (Table 1) were used to amplify the upstream fragment of *pic*, with primers down*pic*-F-XbaI and down*pic*-R-BamHI (Table 1) used to amplify the downstream fragment. The amplified downstream fragment of *pic* was digested with *Xba*I and *Bam*HI and ligated into pSB890 which had been cut with the same restriction endonucleases [27]. We designated the resulting plasmid pSB890-*pic* downstream. The amplified upstream *pic* fragment was digested with *Not*I and *Xba*I and ligated into pSB890-*pic* downstream that had been digested with *Not*I and *Xba*I. The resulting vector was designated pSB890-∆ *pic* and transformed into *E. coli* SM10 λpir cells, then introduced into SF301 through a bacterial conjugation test. After culturing on a sucrose LB agar plate at 22°C, sucrose-tolerant colonies were screened using *Shigella*-specific minimal medium [7] and a PCR employing primers Upup*pic*-F and Downdown*pic*-R (Table 1). The mutant strain with the *pic* deletion was identified by sequencing and named SF301-∆ *pic*.

### Construction of complementation strains SF301*-*∆ *pic*/pPic and SF51/pPic

A plasmid containing *pic* was constructed using pSC modified from pREP4. The *pic* gene was amplified from SF301 genomic DNA using PCR. The PCR primers used were *pic*-pSC-F-PfMlI and *pic*-pSC-R-AclI (Table 1). Amplicons were inserted into pSC, creating pSC*-pic*, which was verified by restriction enzyme digestion and nucleic acid sequencing. Verified pSC-*pic* was transformed into SF301-∆ *pic* and SF51, resulting in SF301-∆ *pic*/pPic and SF51/pPic, respectively.

### *S. flexneri* growth curves

The growth curves of *S. flexneri* 2a strains were determined by measuring the optical density at 600 nm (OD_600_) as described previously [28]. Briefly, overnight cultures were diluted 1:200 and incubated at 37°C with shaking (220 rpm). Samples (1 mL) of the bacterial cultures were taken every 30 min over 16 h and OD measured. Growth curves were created by plotting OD_600_ against incubation time (h).

### *S. flexneri* HeLa cell invasion assays

*S. flexneri* cell invasion assays were used to test the virulence of a SF51 clinical strain without *set1B*, SF301-∆ *pic*, wild-type SF301, SF301-∆ *pic*/pPic and SF51/pPic. The ability of bacteria to invade HeLa cells was determined using a gentamicin protection assays [29]. HeLa cells were grown in 6-well tissue culture plates in DMEM supplemented with 10% FCS and incubated at 37°C/5% CO_2_ until they formed semi-confluent monolayers. SF51, SF301-∆ *pic*, SF301-∆ *pic* /pPic, SF51 /pPic and SF301 were individually added to semi-confluent HeLa cells at an MOI of 100. Bacteria were diluted and plated on LB agar plates. Colony-forming units (CFUs) were counted and added to HeLa cells. Plates were centrifuged at 900 × *g* for 5 min. After incubating at 37°C for 90 min, cells were washed three times with PBS, and gentamicin added to the medium at a final concentration of 10 μg/mL. The mixture was then incubated for 20 min at 37°C. HeLa cells in each well were lysed with 1 mL of PBS containing 0.1% Triton X-100 for 10 min at room temperature. Lysates were diluted and plated onto LB agar plates in triplicate. Colonies that grew on LB plates were counted. Results were expressed as the number of bacteria recovered from gentamicin-treated cells divided by the number of inoculated bacteria added to the cell. Cells inoculated with *E. coli* ATCC 25922, an avirulent strain, were the negative controls. Cell invasion assays were performed in triplicate for each strain, and the assay repeated twice.

### Sereny tests and pathohistological examination

A mouse Sereny test was used to evaluate the virulence of all strains we examined in this study, as described by Murayama [30]. A single red colony of *S. flexneri* on Congo red agar [Tryptic soy broth (Oxoid), 1.5% (w/v) agar and 0.01% (w/v) Congo red] was inoculated into LB broth at 37°C for 8 h with constant shaking. Female BALB/c mice (4–5-weeks-old) were infected with 1 × 10^8^ CFUs per eye (n = 4 eyes, two mice in each group). Symptoms and signs of keratoconjunctivitis in mice infected with bacteria were observed at 24, 48, 72, and 96 h post-inoculation [28,30]. Eyes inoculated with *E. coli* ATCC 25922 and normal saline (NS) served as the negative controls. The invasiveness of bacteria was scored according to the following system: ‘−’ indicates no inflammation, and an infection level score of 0; ‘±’ is indicative of low levels of keratoconjunctivitis, and an infection level score of 0.5; ‘+’ indicates slight conjunctival inflammation with eyelid edema, and an infection level score of 1; ‘++’ indicates mild keratoconjunctivitis with eyelid edema, increased tear film evaporation and periocular hair loss, and an infection level score of 2; and ‘+++’ indicating fully developed keratoconjunctivitis with eyelid swelling, periocular hair loss, blepharophimosis, conjunctival follicles and purulent discharge, and an infection level score of 3. At 24, 48, 72, and 96 h post-inoculation, mice were euthanized and the eyes removed and fixed in 4% formalin in PBS (pH 7.2). After hemotoxylin and eosin (H&E) staining, eye sections were examined using a light microscope.

### Statistical analysis

Experimental data were analyzed with SPSS and comparisons made using Student’s *t*-test. Differences with a *P*-value less than 0.05 were considered statistically significant.

## Results

### Detection of *ipaH*, *ial*, and *set1B* in *S. flexneri* clinical isolates

The *ipaH* gene was detected in all 86 *S. flexneri* clinical isolates, whereas *ial* was detected in 45 isolates (52.3%), and *set1B* detected in 69 isolates (80.2%). Amplicons for *ipaH*, *ial* and *set1B* were not seen from *E. coli* ATCC 25922 samples. All three genes were detected in the SF301 positive control.

### HeLa cell invasion of *S. flexneri* clinical isolates

**Figure 1:**
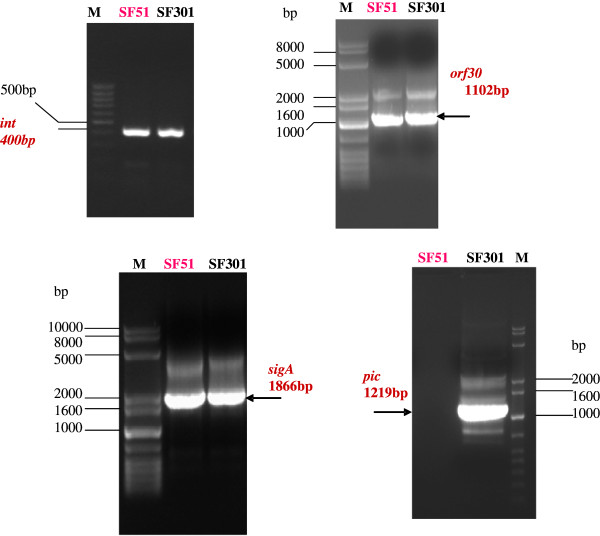
**Amplicons from SF51 *int*, *orf30*, *sigA *and *pic*.** Specific amplicons for (**A**) *int*; (**B**) *orf30*; (**C**) *sigA*; and (**D**) *pic.* The target genes *int*, *orf30* and *sigA* were amplified from SF51 DNA but *pic* was not. All four target genes were amplified from the SF301 positive control. The sequence of all PCR products was confirmed by nucleic acid sequencing.

Nine isolates in our study contained all three genes, one SF68 isolate contained *ipaH* and *set1B*, one SF51 isolate had *ipaH* and *ial*, and one SF36 isolate contained only *ipaH* (Table 2). The nine isolates that contained all three virulence-associated genes demonstrated high invasive ability in HeLa cells (>200 plaques/well). In HeLa cells, SF68 and SF36 were less invasive resulting in 4 and 2 plaques/well, respectively. SF51 lacked *set1B* but retained *ial*, and showed a decrease in invasiveness (20 plaques/well; Table 2). Using PCR and nucleotide sequencing, it was shown that SF51 lacked the entire *pic* gene on PAI-1, but harbored *sigA* and other significant open reading frames (Figure 1).

**Table 2:** **Invasion of HeLa cells by *S. flexneri *clinical isolates as determined by plaque formation tests**

**Gene detected by mPCR**			**Number of strains**	Plaque formation number (per well)*
***ipaH***	***ial***	***set1B***		
+	+	+	9	>200±16
**+**	**+**	**-**	1 (SF51)	20±5
+	**-**	+	1 (SF68)	4±2
**+**	**-**	**-**	1 (SF36)	2±1

* Values represent the mean ± standard deviation of three wells.

### HeLa cell invasion of SF301 and mutants

**Figure 2:**
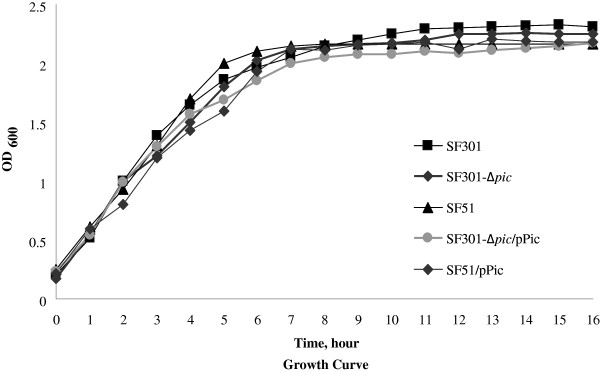
**Growth curves for SF301 and the *pic *mutants (SF51, SF301*-*∆ *pic*, SF301-∆ *pic*/pPic and SF51/pPic).**

**Figure 3:**
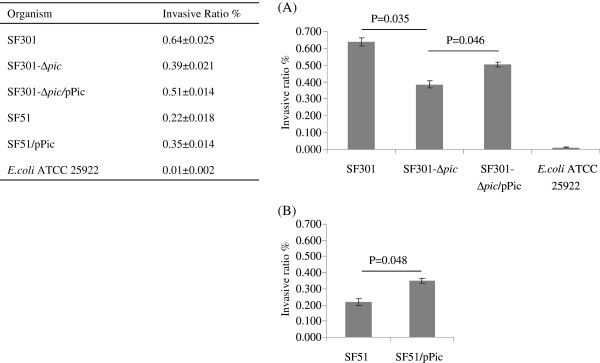
**HeLa cell invasion assays for SF301 and the *pic *mutants.** (**A**) The HeLa cell invasion abilities of SF301, *pic* knockout mutant of SF301 (SF301*-*∆ *pic*), *pic* complementation of SF301*-*∆ *pic* (SF301-∆ *pic*/pPic) and *E. coli* ATCC 25922. (**B**) The invasion abilities of *pic* complementation of SF51 (SF51/pPic) compared with clinical isolate SF51. Values are presented as mean ± SD.

The growth curves for SF51, SF301*-*∆ *pic*, SF301, SF301-∆ *pic*/pPic and SF51/pPic were similar under aerobic growth conditions (Figure 2). Gentamicin protection tests on HeLa cells revealed that the cell invasion ratio for clinical isolate SF51 with standard strain SF301 decreased by 34%, while the invasion ratio for SF301-∆ *pic* compared with SF301 decreased by 61% (Figure 3A). The invasion abilities were partially recovered by the introduction of *pic* into deleted mutant SF301-∆ *pic*, which increased the ratio by 31% (to a final cell invasion ratio of 51%, Figure 3A). The invasion abilities of SF51/pPic increased by 59% compared with SF51, with cell invasion ratios of 35% and 22%, respectively (Figure 3B). The *E. coli* ATCC 25922 strain was not found to invade HeLa cells.

### Mouse Sereny tests and pathohistological examination

**Figure 4:**
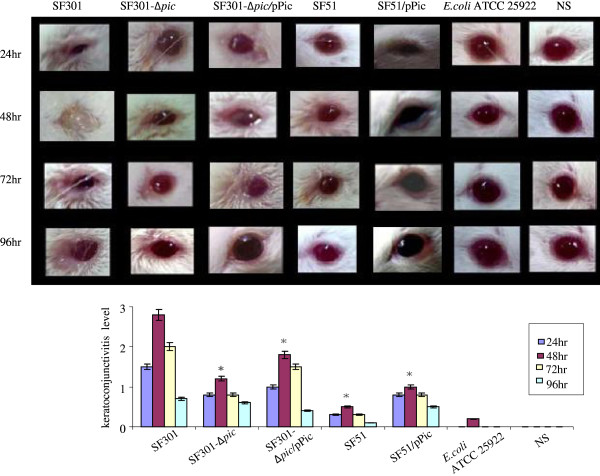
Images of keratoconjunctivitis from mouse Sereny tests for SF301 and *pic *mutants. *P < 0.05 vs. SF301.

Mouse Sereny tests confirmed the results of the cell invasion tests. Mild presentation of keratoconjunctivitis was observed 24 h after mice were infected with SF301. Symptoms included eyelid edema, increased tear film evaporation and periocular hair-loss that we scored as either + or ++, with an average infection level score of 1.5. This developed into severe keratoconjunctivitis with maximal blepharophimosis at 48 h that we rated +++, and an average infection level score of 2.8. Keratoconjunctival inflammation continued for 96 h post-inoculation with SF301 (Figure 4). Both the isolated and constructed *pic*-deletion mutants induced lower levels of inflammation in the eyes of mice than for SF301 (Figure 4). At 48 h post-inoculation, the pathogenicity of SF301-∆ *pic* in mouse eyes were assessed as + or ++ with an average infection level scores up to 1.2; for SF51, pathogenicity was rated ± or + with an average infection level score less than 0.6.

Virulence was partially recovered by introducing the complementary pSC-*pic* into the deletion mutants. At 48 h post-inoculation the pathogenicity of SF301-∆ *pic*/pPic was rated at + or ++ with an average infection level score 1.9; SF51/pPic pathogenicity was + or ++ with average infection level scores of 1.2. At 48 h post-infection, inflammatory reactions were not observed in the normal saline negative controls (−, 0). However, *E. coli* ATCC 25922 slight edema (±) in a single eyelid at 48 h post-infection with an average infection level score of 0.3.

**Figure 5:**
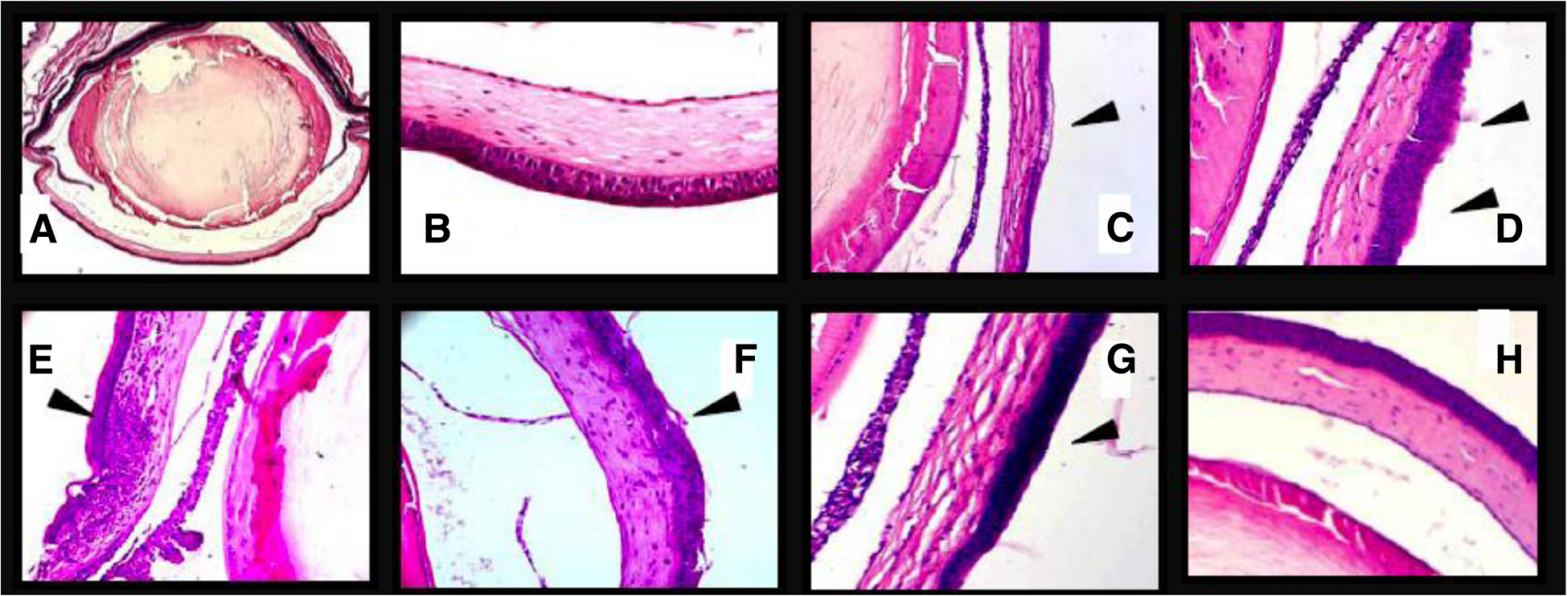
**Histological features of mouse corneas following Sereny tests with SF301 and *pic *mutants. (A, B)** Following inoculation with normal saline, normal corneal epithelium with many layers arranged in an orderly manner can be seen (A: ×50 magnification; B ×400 magnification). **(C)** After infection with SF301, the corneal epithelium was thinner than that of the control, and vesicular changes (arrowheads) were observed (×100 magnification). **(D)** Corneal epithelial edema was observed (arrowheads; ×200 magnification). **(E)** Polymorphic nuclear neutrophilic activity was observed (arrowheads; ×200 magnification). **(F)** Corneal epithelial derangement and detachment were observed (arrowheads; ×200 magnificaiton). **(G)** After infection with SF301-∆ *pic* little damage was observed, but corneal epithelial hyperplasia was noted (arrowheads; ×200 magnification). **(H)** After infection with SF51, little damage was observed (×200 magnification).

Light microscopy assessment at 48 h post-infection revealed typical symptoms of SF301 infection. These included limited invasion, corneal epithelial thickening and loss, along with mild, moderate, or severe ulcers*.* Both *pic-*deletion mutants showed fewer pathologic changes following H&E staining compared with SF301 (Figure 5).

## Discussion

*Shigella* pathogenicity is a multigenic phenomenon involving the participation of genes on the unstable large virulence plasmid and chromosomal PAIs [12-14,17,28,31-34]. Mobile genes encode key factors that help *Shigella* invade tissue and maintain its intracellular viability [13,17,35-38]. The pathogenicity of the strain decreases markedly once the mobile genes are deleted [4,32,33].

Several studies have been conducted to detect virulence genes in *Shigella* by mPCR, targeting *ipaH*, *ial*, and *rfc* or *stx1* for serotype identification [3,5,7,39]. In 2005, Thong [5] first described a new mPCR system to detect *S. flexneri* 2a by targeting four virulence genes (*ipaH*, *ial*, *set1A* and *set1B*). This mPCR system was able to determine, in a single reaction, whether genes related to pathogenesis of a particular *Shigella* strain are associated with the chromosome or plasmid, and whether the serotype of the particular strain can be grouped under *S. flexneri* 2a [4,5]. In our present study, Thong’s mPCR system was modified to identify *S. flexneri* 2a strains and their virulence using only three virulent genes (*ipaH*, *ial*, and *set1B*). We omitted *set1A* from the mPCR system, as both *set1B* and *set1A* genes have been shown to exist in tandem on PAI-1 of the bacterial chromosome, and they share the same promoter [5,21]. The low prevalence of *ial* (45/86, 52.3%) verifies that the cell-entry region on the large virulence plasmid of *S. flexneri* is prone to loss or deletion. The high prevalence of the *set1B* gene (69/86, 80.2%) verifies that in the rural regions of Zhengding, the isolated epidemic strain of *Shigella* was *S. flexneri* 2a. All of our mPCR results were confirmed by serological tests. We confirmed that comparable decreases in virulence occur following the deletion of essential elements in the large virulence plasmid (*ipaH* and *set1B* for SF68; and *ipaH* for SF36) [35-38]. A clinical SF51 isolate was found to retain *ial* but had lost *set1B*, and demonstrated an obvious decrease in HeLa cell invasion. This indicated to us that the chromosome locus around *set1B* may influence virulence.

The location of *set1B* is known to be in *Shigella* PAI-1 [7,20], which exists exclusively in *S. flexneri* 2a*.* At least four major virulence genes are present in PAI-1 (*pic*, *set1A*, *set1B*, and *sigA*). The autotransporter SigA exhibits cytopathic effects on HEp-2 cells [40], and the autotransporter Pic exhibits hemagglutination and mucinolytic activities *in vitro*[20-23,41-43]. Upstream from *pic* are two IS elements, IS*911* and IS*629*, followed by *pic* itself, and then a *perD* IS element [21]. This implies that *pic* can be spontaneously deleted.

The upstream element *int*, downstream element *orf30*, cytopathic factor gene *sigA*, and the hemagglutinin gene *pic* on PAI-1 of SF51 were sequenced to verify whether SF51 lost the whole PAI-1 or only part of the genetic locus around *set1B*. Our results revealed that the entire *pic* gene on PAI-1 was deleted in this case, whereas other genes (*sigA*, *int*, and *orf*30) were unaffected (Figure 1). This result also suggests that a decrease in virulence of SF51 is not related to *sigA*, but may be associated with *pic* deletion.

To confirm that the decreased virulence phenotype in SF51 was associated with deletion of *pic*, we knocked out *pic* from the SF301 strain to produce SF301-∆ *pic*. Additionally, complementation strains SF301-∆ *pic*/pPic and SF51*pic*/pPic were constructed to demonstrate that the decreased virulence of SF51 was associated with the deletion of *pic*. Using gentamicin protection assays, we showed that the Hela cell invasion potential of the *pic* knockout strains, SF51 and SF301-∆ *pic*, was decreased compared with the wild-type SF301 strain. This decreased virulence was partially recovered by introducing pSC-*pic*.

Previous studies have demonstrated that purified recombinant protein Pic (prepared from *E.coli* HB101 (pPic1)) is not involved in cytotoxic effects on HT29-C1 and HEp-2 cells [24,25]. However, the findings from our current study show that both the clinical and constructed *pic*-deleted mutants possessed a decreased tendency for cell invasion compared with SF301. Virulence was partially recovered through the insertion of a complementary *pic* gene into these deletion mutants. Because Pic did not elicit cytopathic effects on epithelial cells, it may be associated with a less efficient interaction process with host cells, lacking any assistance from bacterial effectors. This phenomenon has also been observed by Vidal et al. [44], who examined the EPEC autotransporter EspC. Purified EspC requires a higher concentration (300 μg/ml vs. 50 μg/ml for other autotransporter cytotoxins) and a longer incubation time (8 h vs. 1 h for EPEC host cells) to produce the same cytotoxic effects as other EPEC isolates. Further studies have confirmed that EspC translocation into epithelial cells results in cytopathic effects in HeLa cells, but require participation of types III and V secretion systems. The mechanism by which Pic is interacted with epithelial cells remains unknown and warrants further study. Further, differences in results observed with Pic regarding decreases in cytopathic effects are likely also associated with other cell lines. Differences in invasion efficiency between Hela cells and HEp-2 cells have been observed for *Streptococcus pyrogenes*, *Campylobacter jejuni* and *Salmonella typhimurium*[45-47]; however, the reasons for these differences remain unclear, and further study is required to clarify this.

The mouse Sereny test is commonly used to the test the invasiveness of *Shigella*[30]. In our work, the virulence of SF51 and SF301-∆ *pic* was obviously decreased. This was partially recovered by the introduction of pSC-*pic* into deletion mutants. Our findings support the conclusion that *pic* is associated with the invasion potential of *S. flexneri* 2a.

Harrington et al. [42] used a mouse model treated with streptomycin to show that Pic promotes intestinal colonization by comparing intestinal colonization abilities of wild-type *E. coli* 042 and *pic* mutants (*E. coli* 042 *pic::aph3* and *E. coli* 042PicS258A). They demonstrated that the constructed mutants (*E. coli* 042 *pic::aph3* and *E. coli* 042PicS258A) contained significant defects that adversely affected colonization of mice gastrointestinal tracts compared with *E. coli* 042. Further work by Harrington et al. suggested that a possible mechanism of promoting intestinal colonization depended on the mucinase activity of Pic. They also showed that this effect is associated with the serine protease catalytic residue in Pic. The research of Harrington et al. supports our findings that Pic is involved in bacterial invasion ability. Whether a decrease in virulence is associated with the mucinase activity of Pic, or other biological activities, should be investigated further.

## Conclusions

Our findings suggest that *pic*, located on PAI-1 of *S. flexneri* 2a, plays a role in cell invasion during *Shigella* infections. Further work is necessary to elucidate how Pic affects host-pathogen interactions, and how Pic assists *S. flexneri* 2a to invade intestinal epithelial cells and cause cytopathic effects.

## Competing interests

The authors declare that they have no competing interests.

## Authors’ contributions

JZ performed the molecular genetic studies, participated in sequence analysis, constructed the *pic* gene deletion mutant and pic gene complementation strains, carried out mouse Sereny tests and drafted the manuscript. XC participated in mouse Sereny tests and conducted H&E staining. XL conducted mPCR tests and performed HeLa cell gentamicin protection assays. LQ and YW participated in the design of the study, performed statistical analysis and edited the manuscript. DQ and YW participated in the design and coordination of the study, and helped to draft and edit the manuscript. All authors read and approved the final version of the manuscript.

## References

[B1] Kotloff KL Winickoff JP Ivanoff B Clemens JD Swerdlow DL Sansonetti PJ Adak GK Levine MM Global burden of Shigella infections: implications for vaccine development and implementation of control strategies Bull World Health Organ 1999 77 8 651 666 10516787 PMC2557719

[B2] Wang XY Tao F Xiao D Lee H Deen J Gong J Zhao Y Zhou W Li W Shen B Trend and disease burden of bacillary dysentery in China (1991–2000) Bull World Health Organ 2006 84 7 561 568 10.2471/BLT.05.023853 16878230 PMC2627389

[B3] Melito PL Woodward DL Munro J Walsh J Foster R Tilley P Paccagnella A Isaac-Renton J Ismail J Ng LK A novel Shigella dysenteriae serovar isolated in Canada J Clin Microbiol 2005 43 2 740 744 10.1128/JCM.43.2.740-744.2005 15695673 PMC548111

[B4] Schroeder GN Hilbi H Molecular pathogenesis of Shigella spp.: controlling host cell signaling, invasion, and death by type III secretion Clin Microbiol Rev 2008 21 1 134 156 10.1128/CMR.00032-07 18202440 PMC2223840

[B5] Thong KL Hoe SL Puthucheary SD Yasin RM Detection of virulence genes in Malaysian Shigella species by multiplex PCR assay BMC Infect Dis 2005 5 8 10.1186/1471-2334-5-8 15707504 PMC551607

[B6] Vargas M Gascon J Jimenez De Anta MT Vila J Prevalence of Shigella enterotoxins 1 and 2 among Shigella strains isolated from patients with traveler’s diarrhea J Clin Microbiol 1999 37 11 3608 3611 10523561 10.1128/jcm.37.11.3608-3611.1999 PMC85705

[B7] Rajakumar K Sasakawa C Adler B Use of a novel approach, termed island probing, identifies the Shigella flexneri she pathogenicity island which encodes a homolog of the immunoglobulin A protease-like family of proteins Infect Immun 1997 65 11 4606 4614 9353040 10.1128/iai.65.11.4606-4614.1997 PMC175661

[B8] Okuda J Toyotome T Kataoka N Ohno M Abe H Shimura Y Seyedarabi A Pickersgill R Sasakawa C Shigella effector IpaH9.8 binds to a splicing factor U2AF(35) to modulate host immune responses Biochem Biophys Res Commun 2005 333 2 531 539 10.1016/j.bbrc.2005.05.145 15950937

[B9] Toyotome T Suzuki T Kuwae A Nonaka T Fukuda H Imajoh-Ohmi S Toyofuku T Hori M Sasakawa C Shigella protein IpaH(9.8) is secreted from bacteria within mammalian cells and transported to the nucleus J Biol Chem 2001 276 34 32071 32079 10.1074/jbc.M101882200 11418613

[B10] Fernandez-Prada CM Hoover DL Tall BD Hartman AB Kopelowitz J Venkatesan MM Shigella flexneri IpaH(7.8) facilitates escape of virulent bacteria from the endocytic vacuoles of mouse and human macrophages Infect Immun 2000 68 6 3608 3619 10.1128/IAI.68.6.3608-3619.2000 10816519 PMC97650

[B11] Rohde JR Breitkreutz A Chenal A Sansonetti PJ Parsot C Type III secretion effectors of the IpaH family are E3 ubiquitin ligases Cell Host Microbe 2007 1 1 77 83 10.1016/j.chom.2007.02.002 18005683

[B12] Sansonetti PJ Kopecko DJ Formal SB Involvement of a plasmid in the invasive ability of Shigella flexneri Infect Immun 1982 35 3 852 860 6279518 10.1128/iai.35.3.852-860.1982 PMC351125

[B13] Sasakawa C Kamata K Sakai T Murayama SY Makino S Yoshikawa M Molecular alteration of the 140-megadalton plasmid associated with loss of virulence and Congo red binding activity in Shigella flexneri Infect Immun 1986 51 2 470 475 3002985 10.1128/iai.51.2.470-475.1986 PMC262355

[B14] Buchrieser C Glaser P Rusniok C Nedjari H D’Hauteville H Kunst F Sansonetti P Parsot C The virulence plasmid pWR100 and the repertoire of proteins secreted by the type III secretion apparatus of Shigella flexneri Mol Microbiol 2000 38 4 760 771 10.1046/j.1365-2958.2000.02179.x 11115111

[B15] Yang F Yang J Zhang X Chen L Jiang Y Yan Y Tang X Wang J Xiong Z Dong J Genome dynamics and diversity of Shigella species, the etiologic agents of bacillary dysentery Nucleic Acids Res 2005 33 19 6445 6458 10.1093/nar/gki954 16275786 PMC1278947

[B16] Jin Q Yuan Z Xu J Wang Y Shen Y Lu W Wang J Liu H Yang J Yang F Genome sequence of Shigella flexneri 2a: insights into pathogenicity through comparison with genomes of Escherichia coli K12 and O157 Nucleic Acids Res 2002 30 20 4432 4441 10.1093/nar/gkf566 12384590 PMC137130

[B17] Venkatesan MM Goldberg MB Rose DJ Grotbeck EJ Burland V Blattner FR Complete DNA sequence and analysis of the large virulence plasmid of Shigella flexneri Infect Immun 2001 69 5 3271 3285 10.1128/IAI.69.5.3271-3285.2001 11292750 PMC98286

[B18] Noriega FR Liao FM Formal SB Fasano A Levine MM Prevalence of Shigella enterotoxin 1 among Shigella clinical isolates of diverse serotypes J Infect Dis 1995 172 5 1408 1410 10.1093/infdis/172.5.1408 7594690

[B19] Fasano A Noriega FR Liao FM Wang W Levine MM Effect of shigella enterotoxin 1 (ShET1) on rabbit intestine in vitro and in vivo Gut 1997 40 4 505 511 9176079 10.1136/gut.40.4.505 PMC1027126

[B20] Al-Hasani K Rajakumar K Bulach D Robins-Browne R Adler B Sakellaris H Genetic organization of the she pathogenicity island in Shigella flexneri 2a Microb Pathog 2001 30 1 1 8 10.1006/mpat.2000.0404 11162180

[B21] Henderson IR Czeczulin J Eslava C Noriega F Nataro JP Characterization of pic, a secreted protease of Shigella flexneri and enteroaggregative Escherichia coli Infect Immun 1999 67 11 5587 5596 10531204 10.1128/iai.67.11.5587-5596.1999 PMC96930

[B22] Henderson IR Nataro JP Virulence functions of autotransporter proteins Infect Immun 2001 69 3 1231 1243 10.1128/IAI.69.3.1231-1243.2001 11179284 PMC98013

[B23] Yen YT Kostakioti M Henderson IR Stathopoulos C Common themes and variations in serine protease autotransporters Trends Microbiol 2008 16 8 370 379 10.1016/j.tim.2008.05.003 18595714

[B24] Gutierrez-Jimenez J Arciniega I Navarro-Garcia F The serine protease motif of Pic mediates a dose-dependent mucolytic activity after binding to sugar constituents of the mucin substrate Microb Pathog 2008 45 2 115 123 10.1016/j.micpath.2008.04.006 18538533

[B25] Dutta PR Cappello R Navarro-Garcia F Nataro JP Functional comparison of serine protease autotransporters of enterobacteriaceae Infect Immun 2002 70 12 7105 7113 10.1128/IAI.70.12.7105-7113.2002 12438392 PMC133081

[B26] Oaks EV Wingfield ME Formal SB Plaque formation by virulent Shigella flexneri Infect Immun 1985 48 1 124 129 3884506 10.1128/iai.48.1.124-129.1985 PMC261924

[B27] Hapfelmeier S Ehrbar K Stecher B Barthel M Kremer M Hardt WD Role of the Salmonella pathogenicity island 1 effector proteins SipA, SopB, SopE, and SopE2 in Salmonella enterica subspecies 1 serovar Typhimurium colitis in streptomycin-pretreated mice Infect Immun 2004 72 2 795 809 10.1128/IAI.72.2.795-809.2004 14742523 PMC321604

[B28] Cai X Zhang J Chen M Wu Y Wang X Chen J Shen X Qu D Jiang H The effect of the potential PhoQ histidine kinase inhibitors on Shigella flexneri virulence PLoS One 2011 6 8 e23100 10.1371/journal.pone.0023100 21853073 PMC3154276

[B29] Yu J Inactivation of DsbA, but not DsbC and DsbD, affects the intracellular survival and virulence of Shigella flexneri Infect Immun 1998 66 8 3909 3917 9673279 10.1128/iai.66.8.3909-3917.1998 PMC108449

[B30] Murayama SY Sakai T Makino S Kurata T Sasakawa C Yoshikawa M The use of mice in the Sereny test as a virulence assay of shigellae and enteroinvasive Escherichia coli Infect Immun 1986 51 2 696 698 3510985 10.1128/iai.51.2.696-698.1986 PMC262412

[B31] Pupo GM Lan R Reeves PR Multiple independent origins of Shigella clones of Escherichia coli and convergent evolution of many of their characteristics Proc Natl Acad Sci USA 2000 97 19 10567 10572 10.1073/pnas.180094797 10954745 PMC27065

[B32] Yang J Nie H Chen L Zhang X Yang F Xu X Zhu Y Yu J Jin Q Revisiting the molecular evolutionary history of Shigella spp J Mol Evol 2007 64 1 71 79 10.1007/s00239-006-0052-8 17160643

[B33] Lan R Reeves PR Escherichia coli in disguise: molecular origins of Shigella Microbes Infect 2002 4 11 1125 1132 10.1016/S1286-4579(02)01637-4 12361912

[B34] Zurawski DV Mumy KL Faherty CS McCormick BA Maurelli AT Shigella flexneri type III secretion system effectors OspB and OspF target the nucleus to downregulate the host inflammatory response via interactions with retinoblastoma protein Mol Microbiol 2009 71 2 350 368 10.1111/j.1365-2958.2008.06524.x 19017275 PMC2783611

[B35] Blocker A Gounon P Larquet E Niebuhr K Cabiaux V Parsot C Sansonetti P The tripartite type III secreton of Shigella flexneri inserts IpaB and IpaC into host membranes J Cell Biol 1999 147 3 683 693 10.1083/jcb.147.3.683 10545510 PMC2151192

[B36] Buysse JM Stover CK Oaks EV Venkatesan M Kopecko DJ Molecular cloning of invasion plasmid antigen (ipa) genes from Shigella flexneri: analysis of ipa gene products and genetic mapping J Bacteriol 1987 169 6 2561 2569 3294797 10.1128/jb.169.6.2561-2569.1987 PMC212123

[B37] Menard R Sansonetti P Parsot C The secretion of the Shigella flexneri Ipa invasins is activated by epithelial cells and controlled by IpaB and IpaD EMBO J 1994 13 22 5293 5302 7957095 10.1002/j.1460-2075.1994.tb06863.x PMC395485

[B38] Menard R Sansonetti PJ Parsot C Nonpolar mutagenesis of the ipa genes defines IpaB, IpaC, and IpaD as effectors of Shigella flexneri entry into epithelial cells J Bacteriol 1993 175 18 5899 5906 8376337 10.1128/jb.175.18.5899-5906.1993 PMC206670

[B39] Gaudio PA Sethabutr O Echeverria P Hoge CW Utility of a polymerase chain reaction diagnostic system in a study of the epidemiology of shigellosis among dysentery patients, family contacts, and well controls living in a shigellosis-endemic area J Infect Dis 1997 176 4 1013 1018 10.1086/516531 9333160

[B40] Al-Hasani K Henderson IR Sakellaris H Rajakumar K Grant T Nataro JP Robins-Browne R Adler B The sigA gene which is borne on the she pathogenicity island of Shigella flexneri 2a encodes an exported cytopathic protease involved in intestinal fluid accumulation Infect Immun 2000 68 5 2457 2463 10.1128/IAI.68.5.2457-2463.2000 10768931 PMC97446

[B41] Navarro-Garcia F Gutierrez-Jimenez J Garcia-Tovar C Castro LA Salazar-Gonzalez H Cordova V Pic, an autotransporter protein secreted by different pathogens in the Enterobacteriaceae family, is a potent mucus secretagogue Infect Immun 2010 78 10 4101 4109 10.1128/IAI.00523-10 20696826 PMC2950354

[B42] Harrington SM Sheikh J Henderson IR Ruiz-Perez F Cohen PS Nataro JP The Pic protease of enteroaggregative Escherichia coli promotes intestinal colonization and growth in the presence of mucin Infect Immun 2009 77 6 2465 2473 10.1128/IAI.01494-08 19349428 PMC2687332

[B43] Ruiz-Perez F Wahid R Faherty CS Kolappaswamy K Rodriguez L Santiago A Murphy E Cross A Sztein MB Nataro JP Serine protease autotransporters from Shigella flexneri and pathogenic Escherichia coli target a broad range of leukocyte glycoproteins Proc Natl Acad Sci USA 2011 108 31 12881 12886 10.1073/pnas.1101006108 21768350 PMC3150873

[B44] Vidal JE Navarro-Garcia F EspC translocation into epithelial cells by enteropathogenic Escherichia coli requires a concerted participation of type V and III secretion systems Cell Microbiol 2008 10 10 1975 1986 10.1111/j.1462-5822.2008.01181.x 18547338

[B45] Greco R De Martino L Donnarumma G Conte MP Seganti L Valenti P Invasion of cultured human cells by Streptococcus pyogenes Res Microbiol 1995 146 7 551 560 10.1016/0923-2508(96)80561-4 8577996

[B46] Prasad KN Dhole TN Ayyagari A Adherence, invasion and cytotoxin assay of Campylobacter jejuni in HeLa and HEp-2 cells J Diarrhoeal Dis Res 1996 14 4 255 259 9203788

[B47] Baumler AJ Tsolis RM Heffron F Contribution of fimbrial operons to attachment to and invasion of epithelial cell lines by Salmonella typhimurium Infect Immun 1996 64 5 1862 1865 8613405 10.1128/iai.64.5.1862-1865.1996 PMC174006

